# Abcès cérébraux révélant une trilogie de Fallot chez un enfant: à propos d’un cas au CHU de Parakou, Bénin

**DOI:** 10.11604/pamj.2019.34.189.20282

**Published:** 2019-12-10

**Authors:** Falilatou Agbeille Mohamed, Gérard Médétinmè Kpanidja, Alphonse Noudamadjo, Serges Hugues Mahougnon Dohou, Kofi Mensa Savi de Tove, Joseph Agossou, Julien Didier Adedemy

**Affiliations:** 1 Faculté de Médecine, Département Mère Enfant, Université de Parakou, Parakou, Bénin; 2 Faculté de Médecine, Département de Médecine et Spécialités Médicales, Université de Parakou, Parakou, Bénin

**Keywords:** Abcès cérébral, trilogie de Fallot, enfant, Bénin, Cerebral abscess, trilogy of Fallot, child, Benin

## Abstract

Les abcès cérébraux sont pourvoyeurs d'une importante morbidité chez les patients atteints de cardiopathie cyanogène. Dans les pays à ressources limitées leur prise en charge est difficile et leur pronostic réservé. Nous rapportons ici un cas d'abcès cérébraux révélant une forme rare de cardiopathie cyanogène, la trilogie de Fallot à Parakou au Nord du Bénin. Il s'est agi d'un garçon de 9 ans, référé d'un hôpital primaire pour une hémiparésie gauche. L'interrogatoire et l'examen physique avaient permis de retrouver une symptomatologie évoluant depuis deux mois faite de céphalées intenses, de fièvre, de vomissements et d'une impotence fonctionnelle de l'hémicorps gauche. Un état général altéré, une cyanose généralisée; une hémiparésie gauche, un souffle systolique au foyer pulmonaire. Le scanner cérébral avait montré des abcès en région pariétale droite et temporale gauche et une hydrocéphalie. L'écho-doppler cardiaque avait montré une sténose pulmonaire serrée, une communication interauriculaire et une hypertrophie ventriculaire droite. Une antibiothérapie faite de ceftriaxone de gentamycine et métronidazole avait été démarrée en urgence et l'indication chirurgicale d'une trépano-ponction posée mais n'a pu être réalisée car l'évolution avait été rapidement défavorable. Les abcès cérébraux constituent une complication classique des cardiopathies cyanogènes. L'issue est fatale en l'absence de prise en charge adéquate d'où l'intérêt d'un diagnostic et d'une prise en charge précoces de ces cardiopathies.

## Abstract (en)

Brain abscesses can cause significant morbidity in patients with cyanogen heart disease. In countries with limited resources treatment, it is difficult and prognosis is guarded. Here we report a case of brain abscesses revealing a rare form of cyanogen heart disease, the trilogy of Fallot, in Parakou in the north of Benin. The study involved a 9-year old boy, referred to a primary hospital for left hemiparesis. Interview and physical examination revealed symptoms evolving for two months including intense headaches, fever, vomiting and functional impairment of the left hemi-corps, altered general state, generalized cyanosis, left hemiparesis, pulmonary systolic murmur. Brain CT scan showed abscesses in the right parietal region and in the left temporal region as well as hydrocephalus. Doppler echocardiography showed stiff pulmonary stenosis, atrial septal defects and right ventricular hypertrophy. Antibiotic therapy including ceftriaxone, gentamicin and metronidazole was started in emergency. Indication for surgical intervention included trepano-puncture but this could not be performed due to rapid unfavorable outcome. Brain abscesses are a common complication of cyanogen heart disease. Outcome is fatal in the absence of adequate management, hence the role of diagnosis and early treatment of these heart diseases.

## Introduction

Les abcès cérébraux constituent une cause importante de morbidité chez les patients atteints de cardiopathie congénitale cyanogène [1]. Cette complication classique est la conséquence de la polyglobulie observée chez ces patients, responsable d'une hyperviscosité sanguine, favorisant des stases qui constituent une zone propice au développement des emboles septiques [2]. Dans nos pays à ressources limitées, les abcès cérébraux posent plusieurs problèmes sur les plans diagnostique, thérapeutique et pronostique. Nous rapportons ici un cas d'abcès cérébraux révélant une trilogie de Fallot chez un garçon de 9 ans.

## Patient et observation

Il s'est agi d'un grand garçon de 9 ans, référé d'un hôpital primaire pour une hémiparésie gauche. L'interrogatoire avait retrouvé une symptomatologie évoluant depuis deux mois faite de céphalées intenses associées à une fièvre non chiffrée, des vomissements faciles et l'installation progressive d'une impotence fonctionnelle des membres thoracique et pelvien gauches. Dans ses antécédents, on avait retrouvé une absence d'échographie anténatale, une notion d'essoufflement lors des tétées, et une dyspnée d'effort d'aggravation croissante depuis la petite enfance ayant nécessité plusieurs consultations dans des formations sanitaires primaires sans diagnostic. L'examen physique à l'admission avait objectivé: un état général altéré, une température à 378°C, une fréquence cardiaque de 82cpm, une fréquence respiratoire de 31cpm, une saturation en oxygène de 74% à l'air ambiant, une cyanose généralisée et un hippocratisme digital. L'examen neurologique avait noté une hémiparésie gauche associée à une paralysie faciale gauche.

**Figure 1:**
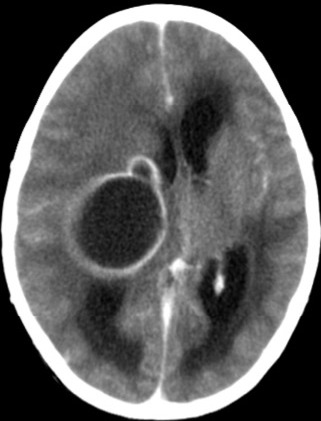
Volumineux abcès avec effet de masse et hydrocéphalie

**Figure 2:**
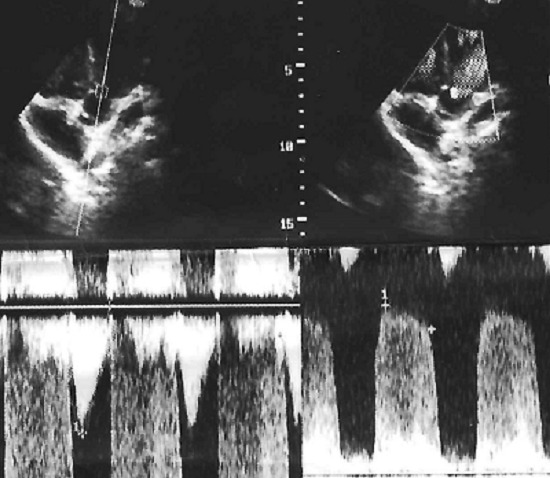
Hyperthrophie auriculaire droite

**Figure 3:**
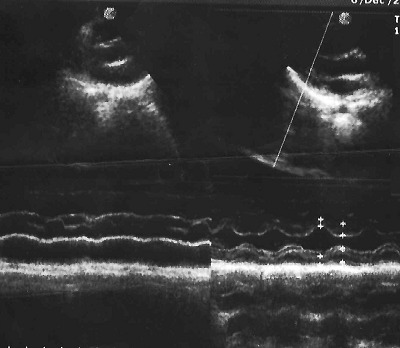
Communication interatriale (CIA) avec shunt droite gauche et hypertrophie auriculoventriculaire

L'examen cardiovasculaire avait retrouvé un souffle systolique d'environ 3/6 siégeant au foyer pulmonaire irradiant faiblement en région axillaire gauche et dans le dos. L'hémogramme avait révélé une polyglobulie avec un taux d'hémoglobine à 19,7g/dl, un hématocrite à 53%, une hyperleucocytose à 22000/mm^3^ à prédominance polynucléaire neutrophile (65%) et les plaquettes à 252000/mm^3^. La créatininémie était normale à 7mg/l. Le scanner cérébral sans et avec injection du produit de contraste avait montré un volumineux abcès pariétal droit de 76x50x45mm soit un volume de 85ml avec effet de masse importante et hydrocéphalie et avec un deuxième abcès en fosse temporale gauche. Il présentait aussi une ectasie des cornes temporales avec effacement des citernes de la base signant une hypertension intracrânienne (Figure 1). Le télécœur avait objectivé une hypertrophie ventriculaire droite avec un indice cardiothoracique à 0,55. L'écho-doppler cardiaque a révélé une sténose pulmonaire serrée avec un gradient entre ventricule droit et artère pulmonaire de 72mmHg; une communication interauriculaire responsable d'un shunt droite gauche, une hypertrophie ventriculaire droite et une dilatation de l'oreillette droite faisant évoquer une trilogie de Fallot (Figure 2, Figure 3). L'ensemble de ces éléments cliniques et para cliniques avait permis d'évoquer le diagnostic d'abcès cérébraux compliquant une trilogie de Fallot. L'enfant avait reçu d'une triple antibiothérapie faite de ceftriaxone à 100mg/Kg/ par jour, de la gentamycine à 5mg/Kg/J et du métronidazole 40mg/kg/ par jour pendant 5 jours. La prise en charge chirurgicale qui devait consister à une trépano-ponction n'avait pu être faite; l'enfant étant décédé avant l'intervention dans un contexte d'hypoxie très sévère.

## Discussion

Les cardiopathies congénitales cyanogènes constituent un facteur prédisposant à la survenue d'abcès cérébraux. L'incidence des abcès cérébraux chez les patients porteurs de cardiopathie cyanogène varie de 5 à 18,7% [3]. Le risque de développer un abcès cérébral survient après l'âge de deux ans et augmente jusqu'à l'âge de 12 ans, puis diminue ensuite avec un pic de fréquence se situant entre 4 ans et 7 ans [4, 5]. Notre patient était âgé de 9 ans, avait eu des contacts avec des formations sanitaires périphériques mais n'avait pas été dépisté précocement pour sa cardiopathie. C'est la survenue de l'abcès cérébral qui a constitué la circonstance de découverte de sa cardiopathie. L'imagerie réalisée dans notre contexte s'est résumée à la tomodensitométrie cérébrale qui a une sensibilité de 95 à 99% dans le diagnostic de l'abcès cérébral [6]. Mais l'imagerie par résonnance magnétique est l'examen par excellence pour le diagnostic des abcès cérébraux. Le diagnostic de la cardiopathie congénitale a été posé sur la base des arguments cliniques, confirmé par l'écho-doppler qui a mis en évidence la sténose pulmonaire serrée associée à une communication interauriculaire et une hypertrophie ventriculaire droite faisant évoquer une trilogie de Fallot.

La trilogie de Fallot fait partie avec la tétralogie de Fallot du groupe des maladies bleues. C'est une forme relativement rare de cardiopathie congénitale cyanogène dont l'incidence n'est pas connue. Les conséquences physiologiques de la sténose pulmonaire avec une communication interauriculaire dépendent du degré d'obstruction à la sortie du ventricule droit ou de l'artère pulmonaire et de la taille de la communication interauriculaire. Elles peuvent être également influencées par la différence de pression diastolique entre le ventricule droit et le ventricule gauche. La sténose de l'orifice pulmonaire conditionne l'anoxie tissulaire et l'hypertrophie ventriculaire droite; celle-ci, augmentant la pression dans l'oreillette droite, entraîne le shunt droite-gauche. Il en résulte une cyanose avec défaillance du ventricule droit. Dans la trilogie de Fallot la symptomatologie se résume principalement à la cyanose qui se développe souvent entre la naissance et l'âge de 12ans. Notre patient se retrouve dans cette tranche d'âge et présentait une cyanose avec une saturation périphérique en oxygène qui n'excédait pas 74%.

D'autres manifestations cliniques peuvent s'y associer: la dyspnée d'effort, les douleurs thoraciques et les syncopes [7]. Sur le plan thérapeutique, la prise en charge médicale a constitué en une triple antibiothérapie reposant sur les céphalosporines de troisième génération, les imidazoles et les aminosides. L'isolement des germes est souvent difficile dans les suppurations intracrâniennes. Les classes d'antibiotiques utilisés dans la prise en charge des abcès cérébraux sont controversées et dépendent de plusieurs facteurs notamment la bactériologie locale souvent rencontrée [8]. Nous avons néanmoins utilisé chez notre patient une antibiothérapie probabiliste ayant une bonne diffusion neuroméningée comme décrit dans la littérature [2, 9]. La prise en charge chirurgicale n'a pu être réalisée chez notre patient au vue de l'évolution rapidement fatale. Selon les auteurs, le pronostic des abcès cérébraux est mauvais dans les cardiopathies cyanogènes. Le taux de mortalité varie entre 27,5 et 71%. Notre patient présentait deux abcès cérébraux et des signes d'hypertension intracrânienne. L'existence d'abcès multiples et d'une hypertension intracrânienne ont été identifiés comme étant des facteurs de mauvais pronostic [9].

## Conclusion

Les abcès cérébraux sont une complication fréquente dans les cardiopathies cyanogènes. L'issue est fatale en l'absence de prise en charge adéquate d'où l'intérêt d'un diagnostic et d'une prise en charge précoces de ces cardiopathies à travers une consultation systématique du nouveau-né et du nourrisson par un personnel soignant qualifié.

## Conflits d’intérêts

Les auteurs ne déclarent aucun conflit d'intérêts.
